# Do physician outcome judgments and judgment biases contribute to inappropriate use of treatments? Study protocol

**DOI:** 10.1186/1748-5908-2-18

**Published:** 2007-06-07

**Authors:** Jamie C Brehaut, Roy Poses, Kaveh G Shojania, Alison Lott, Malcolm Man-Son-Hing, Elise Bassin, Jeremy Grimshaw

**Affiliations:** 1 Ottawa Health Research Institute, Ottawa Hospital, Civic Campus, 1053 Carling Avenue, Ottawa, Ontario, K1Y 4E9, Canada; 2 Department of Epidemiology and Community Medicine, Faculty of Medicine, University of Ottawa, 451 Smyth Road, Ottawa, Ontario, K1H 8M5, Canada; 3 Foundation for Integrity and Responsibility in Medicine, 16 Cutler Street, Suite 104, Warren, RI, 02885, USA; 4 Department of Medicine, Warren Alpert Medical School of Brown University, Providence, RI, 02912, USA; 5 Faculty of Medicine, University of Ottawa, 451 Smyth Road, Ottawa, Ontario, K1H 8M5, Canada; 6 Oral Health Policy and Epidemiology, Harvard School of Dental Medicine, 188 Longwood Avenue, Boston, MA, 02115, USA; 7 Centre for Best Practices, Institute of Population Health, University of Ottawa, 1 Stewart Street, Ottawa, Ontario, K1N 6N5, Canada

## Abstract

**Background:**

There are many examples of physicians using treatments inappropriately, despite clear evidence about the circumstances under which the benefits of such treatments outweigh their harms. When such over- or under- use of treatments occurs for common diseases, the burden to the healthcare system and risks to patients can be substantial. We propose that a major contributor to inappropriate treatment may be how clinicians judge the likelihood of important treatment outcomes, and how these judgments influence their treatment decisions. The current study will examine the role of judged outcome probabilities and other cognitive factors in the context of two clinical treatment decisions: 1) prescription of antibiotics for sore throat, where we hypothesize overestimation of benefit and underestimation of harm leads to over-prescription of antibiotics; and 2) initiation of anticoagulation for patients with atrial fibrillation (AF), where we hypothesize that underestimation of benefit and overestimation of harm leads to under-prescription of warfarin.

**Methods:**

For each of the two conditions, we will administer surveys of two types (Type 1 and Type 2) to different samples of Canadian physicians. The primary goal of the Type 1 survey is to assess physicians' perceived outcome probabilities (both good and bad outcomes) for the target treatment. Type 1 surveys will assess judged outcome probabilities in the context of a representative patient, and include questions about how physicians currently treat such cases, the recollection of rare or vivid outcomes, as well as practice and demographic details. The primary goal of the Type 2 surveys is to measure the specific factors that drive individual clinical judgments and treatment decisions, using a 'clinical judgment analysis' or 'lens modeling' approach. This survey will manipulate eight clinical variables across a series of sixteen realistic case vignettes. Based on the survey responses, we will be able to identify which variables have the greatest effect on physician judgments, and whether judgments are affected by inappropriate cues or incorrect weighting of appropriate cues. We will send antibiotics surveys to family physicians (300 per survey), and warfarin surveys to both family physicians and internal medicine specialists (300 per group per survey), for a total of 1,800 physicians. Each Type 1 survey will be two to four pages in length and take about fifteen minutes to complete, while each Type 2 survey will be eight to ten pages in length and take about thirty minutes to complete.

**Discussion:**

This work will provide insight into the extent to which clinicians' judgments about the likelihood of important treatment outcomes explain inappropriate treatment decisions. This work will also provide information necessary for the development of an individualized feedback tool designed to improve treatment decisions. The techniques developed here have the potential to be applicable to a wide range of clinical areas where inappropriate utilization stems from biased judgments.

## Background

The problem of inappropriate use of existing treatments represents a significant challenge for knowledge translation (KT) researchers. There is mounting evidence that a wide variety of treatments are either under- or over-used, and that this inappropriate use causes significant burden to health-care systems. For example, cardiovascular complications are the most common cause of death among diabetics, yet despite clear evidence of benefit, less than 50% receive angiotensin-converting enzyme (ACE) inhibitors [1]. In contrast, other work has shown that benzodiazepines are over-used, despite clear guidelines that they should be used cautiously [2]. At a more general level, studies from the US and the Netherlands suggest that approximately 30 to 40% of patients do not receive care according to current scientific evidence and approximately 20 to 25% of care provided is either not needed or potentially harmful [3-6].

KT frameworks that characterize the process of translating new evidence into practice change typically recognize the individual practitioner as a key component in the process [7,8]. Indeed, 80% of interventions have focused on the individual practitioner (e.g., continuing medical education, educational outreach, audit and feedback, reminders) [9]. Despite all this research, the options of what interventions to choose, and how to evaluate them, have been driven more by investigator preference than by explicit empirical or theoretical rationale. Any such rationale would need to consider, at a minimum, what is known about how individuals make decisions. The current project will begin the work of applying existing cognitive psychological theory to the problem of changing physician behaviour at the level of the individual practitioner.

### Theoretical basis for physician behaviour change: human judgment and decision making

Most KT frameworks recognize the individual practitioner as a key component in the process of practice change, because it is the practitioner who ultimately makes diagnosis and treatment decisions. This is particularly true in areas where physician autonomy is high, as is the case with many kinds of pharmaceutical treatment. In these situations, it is ultimately the individual practitioner who decides whether or not to prescribe medicines for a patient. In terms of understanding how individuals change their treatment behaviour, one area of psychological theory has been under-utilized. Cognitive psychology, and in particular the judgment and decision-making literature, has developed both theoretical frameworks and methods that could be exploited to develop and improve KT interventions aimed at the individual practitioner [10-12]. The current work hinges on two fundamental claims that have their empirical foundation in the judgment and decision-making literature.

### Claim one: physicians' treatment decisions often depend on their judgments of treatment outcome probabilities

Judgment and decision making psychologists have proposed a variety of models of how people make decisions. These models range from "non-decision" behaviours, performed reflexively and without considering specific case features or alternative courses of action, to the hyper-rational (and unpragmatically complex) tenets of formal decision analysis [13]. Many psychologists now believe that human decision making often falls somewhere between these two extremes. Many decisions will incorporate common elements, such as identifying decision options and their possible outcomes, judging the likelihood and value of these outcomes, and then combining this information to make a decision [13]. Although errors can occur with any of these elements [14], several lines of evidence lead us to study errors in judgments of outcome likelihood, and whether improving such judgments might increase appropriate use of treatments. First, there is considerable evidence showing that physicians have trouble accurately judging the probability of important clinical events and outcomes in a variety of clinical settings [15]. Second, several surveys have also suggested that physicians make decisions about pharmaceutical treatment according to their judgments of the likelihood of relevant outcomes [16]. Third, a pilot study by the authors showed that physicians use their judgments of treatment effectiveness and adverse reaction probabilities to decide upon treatment for congestive heart failure [15]. The two clinical problems selected for this current study involve pharmaceutical treatment decisions and share many characteristics with the pilot study condition. However, we will evaluate whether claim one holds true for these two new clinical situations.

In short, changing physician treatment decisions may rest on improving physicians' judgments of outcome probabilities. One of the goals of this project is to determine whether hypothetical treatment decisions involving two pharmaceutical treatment decisions depend upon these judged outcome probabilities.

### Claim two: cognitive factors can cause errors in physician judgments of treatment outcome probabilities

There is clear evidence that physicians often make errors when making diagnostic or prognostic judgments [17-21], and that individual physicians [22] and groups of physicians [23] vary in their ability to make these judgments. Many of these errors have been attributed to "cognitive biases", which can be defined as the tendency to systematically over- or underestimate particular outcome probabilities. An example of such a tendency is "ego bias", which is the tendency to believe that one's own performance is likely to be better than average [24]. One study showed that ego bias can lead to systematic errors in physicians' prognostic judgments for critically ill patients [4].

In addition to studying systemic errors or biases in the thinking of decision makers, considerable work has focused on cognitive 'heuristics'. These simple mental rules-of-thumb very often produce accurate judgments and are thus highly efficient [25,26]. However, in some situations such shortcuts actually mislead and degrade some diagnostic and prognostic judgments. For example, the "availability heuristic" bases the judgment of a particular outcome probability on the ease with which one can recall instances of similar outcomes [23]. Since vivid events are often more easily recalled than mundane ones, this heuristic could cause one to overestimate the likelihood of unusual or bizarre cases and underestimate the likelihood of more commonplace ones. For example, previous studies have shown that the availability heuristic may affect physicians' diagnostic judgments for bacteremia [23]. One of the goals of the current work is to determine the extent to which cognitive heuristics such as availability contribute to inappropriate use of treatments by physicians.

Some cognitive factors might be expected to affect disproportionately certain subsets of physicians. For example, one study found that the "illusion of control", the tendency to have too much faith in one's own ability to control future events [27,28], can explain why cardiologists generally judge the probabilities of adverse outcomes due to cardiac procedures to be lower than do other internists [29]. Furthermore, less experienced decision makers may be more likely to be influenced by indicators not reliably associated with the outcome. For example, a cracking sound at the time of an ankle injury is unrelated to the presence of a fracture, yet many less experienced emergency physicians report considering this indicator when deciding whether to order radiography [30]. Examination of the extent to which groups of decision makers differ in their assessments of outcome probabilities and their relative susceptibility to different cognitive biases warrants further study.

### Examples of clinical therapies that are inappropriately utilized

This project will examine whether inappropriate treatment decisions are associated with judged outcome probabilities and judgment biases. Two clinical conditions were selected; one in which treatment is generally over-utilized, the other where it is under-utilized. We examine both over- and under-utilization because changing an existing, well-practiced behaviour (i.e. reducing the use of over-utilized treatments) may require different change mechanisms than beginning a new behaviour (i.e. adopting an under-utilized treatment). This proposal focuses on two specific treatments: the over-prescription of antibiotics for pharyngitis treatment, and the under-use of warfarin (Coumadin) for treatment of chronic AF.

Our goal for both clinical conditions is to understand relationships between treatment decisions and judged probabilities of 'outcomes'; i.e. the benefits and harms that might stem from a given treatment. In the case of warfarin treatment for AF, key outcomes will include stroke (fatal or permanently disabling) and major hemorrhages (fatal, intracranial, or other bleeds requiring hospitalization). In the case of antibiotics for pharyngitis, relevant outcomes include resolution of symptoms, local and systemic complications from such infections (e.g., perotonsillar abscess and glomerulonephritis), and complications of treatment, such as adverse drug reactions (ADRs).

### Under-use of warfarin (Coumadin) for treatment of AF

There are many documented examples of physicians failing to use treatments where the benefits clearly outweigh the risks and costs. Such failures to use effective treatments [31-41] can have major implications on health-related costs and overall patient care [6], and guideline developers argue that the detection of instances when physicians fail to use treatments of proven effectiveness should be a cornerstone of quality assessment [42].

One example of an underused effective treatment is anti-coagulation with warfarin (Coumadin) for the treatment of chronic AF. AF is a common cardiac arrhythmia, affecting 5% of the population over the age of 65 [43,44]. While AF increases the risk of stroke six-fold [45,46], use of the anti-coagulant warfarin can substantially reduce that risk [47]. However, there is evidence that despite its effectiveness, anti-coagulants are only taken by 30–60% of appropriate patients. A variety of reasons for this under-use, including those to do with its perceived outcome probabilities by prescribing physicians [48-50], have been proposed but never empirically tested. We will survey samples of family physicians and internal medicine specialists about their practice of prescribing anti-coagulation for people with AF.

### Over-use of antibiotics for sore throat (pharyngitis)

Bacterial resistance to antibiotics has become a global public health problem [51,52]. The over-use of antibiotics by humans is clearly an important cause of this problem [51], much of which can be attributed to the prescribing practices of physicians [52]. One study found that physicians prescribed antibiotics for between 57% and 74% of patients with pharyngitis [53]. Yet, despite the widespread use of antibiotics for pharyngitis, the literature shows very little evidence of the effectiveness of these treatments in terms of speed of symptom resolution or lower rates of adverse events among patients with pharyngitis. While some evidence may demonstrate effectiveness of narrow-spectrum antibiotics among patients with high likelihood of streptococcal pharyngitis [54-56], these benefits do not appear to extend to the wider population of all patients with pharyngitis. Furthermore, the use of broad spectrum antibiotics for pharyngitis may be on the rise, yet there is no evidence of any increased benefit of these antibiotics over more narrow-spectrum choices [53,57,58].

Our review identified four studies that compared cephalosporins to penicillin, all of which showed no benefits [59-62]. Five studies showed no evidence that extended-spectrum macrolides produce any improvement over penicillin V or erythromycin [63-68]. The one study comparing amoxacillin/clavulinic acid to penicillin also failed to show any benefits of the antibiotic [69]. No studies have compared the use of any fluoroquinolone or broad-spectrum antibiotic to penicillin among patients with pharyngitis. In short, the literature on treatment for pharyngitis does not justify use of antibiotics on the general population of patients with pharyngitis, and has failed to uncover any evidence that broad-spectrum antibiotics produce any additional benefit over narrow-spectrum choices like penicillin. Previous interventions to reduce antibiotic use have met with limited success. Some methods involving personalized feedback have been somewhat effective, although these interventions are also labor-intensive, costly and complex, with little known about the extent to which the observed practice change is sustained [70,71].

### Hypotheses

We will examine the role of judged outcome probabilities and judgment biases for two kinds of treatment decisions: use of antibiotics for patients with pharyngitis, and use of anti-coagulants for treatment of AF. The study will address five specific hypotheses:

1. Physicians' decisions to use specific treatments depend on their judgments of the likelihood of treatment outcomes.

2. Physician judgments of the likelihood of treatment outcomes will sometimes be inaccurate;

3. Specific judgment heuristics can account for some of the inaccuracies of physician judgments of treatment outcomes;

4. Predictable groups of physicians will be more apt to be inaccurate in their judgments of treatment outcomes;

5. Judgment inaccuracies will stem from physicians attending to cues that are unrelated to treatment outcomes, and/or insufficiently attending to cues that are related to outcomes.

## Methods

Four surveys will be mailed to Canadian physicians, two focused on the use of antibiotics for pharyngitis, and two on the use of anti-coagulants for treatment of AF. For each clinical condition, one survey (Type 1) will measure the accuracy of judged probabilities of treatment-related outcomes, while the other (Type 2) will use a series of realistic case vignettes to determine what factors affect treatment decisions.

Development of the various surveys will require us to perform the following tasks: systematically review the relevant clinical literatures to identify the characteristics of patients to whom the research results would generalize; identify the important outcomes, good and bad, conditional on treatment; develop evidence-based estimates of the population rates of these outcomes conditional on choice of treatment; and assess the evidence about patient factors that may predict these outcomes. We will also review the available evidence about factors that influence physicians' decisions around use of the treatment. We will construct and pilot test surveys to evaluate physicians' judgments and decisions based on this work. These surveys will be informed by pilot work done in the US on a different range of clinical subspecialties.

### Procedure

The primary goals of the Type 1 surveys will be to assess physicians' perceived outcome probabilities (good and bad) for different treatments, and to compare these perceived probabilities to the real rates indicated by systematic reviews (hypothesis two). These goals will be achieved by having physicians assess a hypothetical patient representative of those included in the most important and relevant RCTs of the target condition. The survey will assess judged outcome probabilities, by asking physicians to quantify the likelihood of various outcomes if a hundred patients similar to this hypothetical patient were to be treated. The Type 1 surveys will also ask physicians about how they currently treat such cases, the recollection of rare or vivid outcomes (hypothesis three), as well as practice and demographic details.

The primary goals of the Type 2 surveys will be to measure specific factors that drive individual clinician judgments and treatment decisions (hypothesis five), and to determine whether individual physician judgments predict treatment decisions (hypothesis one). These goals will be achieved by having physicians consider sixteen realistic case vignettes about hypothetical patients with the target condition. Eight clinical variables will be varied systematically across the sixteen case vignettes using a partial factorial design. For example, the manipulated variables in the antibiotics vignettes could include factors related to clinical outcomes (e.g. Centor criteria predicting strep: cough, fever, tonsillar exudates, tender lymph nodes), as well as non-predictive variables that might be perceived as predictive (e.g. age, sex, occupation). The vignettes will prompt physicians to indicate what management decision they would select for each clinical variable combination. These responses will allow for the identification of which variables have the greatest effect on physician judgments, and whether such judgments are affected by non-predictive cues or the unrealistic expectations of appropriate cues.

Four surveys will be mailed to different random samples of Canadian physicians. The pharyngitis surveys will be administered to different samples of 300 family physicians. Each warfarin survey will be administered to 300 family physicians and 300 internal medicine specialists; this design reflects the fact that this clinical decision is made by both groups of physicians. It will also allow us to examine differences in decision making between two different disciplines (hypothesis four).

We therefore propose to mail four different surveys to a total of 1800 physicians (1200 family physicians and 600 internal medicine specialists). The names, addresses, and telephone numbers of these physicians will be obtained from the Canadian Medical Association Directory and membership lists of specialty organizations, such as the Canadian College of Family Physicians and the Royal College of Physicians and Surgeons of Canada. The sampling population will be limited to English-speaking physicians, since the detailed nature of the surveys would make translation into French extremely time-consuming, requiring a lengthy series of iterations of translation and back-translation to ensure comparability between languages. Random selection from membership lists will result in a sampling population that has approximately the same ratio of physicians from all provinces and territories as in the membership list.

While considerable research has demonstrated the difficulty of obtaining high response rates from physicians, the members of this team have considerable experience in doing so with comparable populations [15,30,72-74]. This project will employ the Dillman Tailored Design Method for survey design and implementation, which is one of the most widely used and tested surveying methods [75]. A recent systematic review demonstrated that recommendations of the Dillman method apply to surveys of physicians [76]. In accordance with the design, an initial pre-notification letter will be sent to all selected physicians and the survey will follow one week later. A series of three reminders and two replacement surveys will then be mailed out to non-responders at two-week intervals. All correspondences will be addressed to the individual physicians, and personally signed by the principal investigator.

The characteristics of the responders and non-responders will be compared, to determine how the generalizability of the survey results may be affected by response bias. This physician-specific information will be obtained from the membership lists used to derive the sampling population. The Dillman method has previously been employed to survey Canadian physician society lists, yielding response rates in excess of 80% [77,78]. The Type 1 surveys will be two to four pages in length and take approximately fifteen minutes to complete. In contrast, the Type 2 surveys will be eight to ten pages in length and take about thirty minutes to complete. There is extensive literature showing that non-trivial financial incentives can improve physician survey response rates anywhere from 8.6% to 48.5% [76]. As a result, a $20 incentive will be offered to all survey participants who return a completed survey.

### Data quality and data collection

Quality assurance procedures will be implemented to ensure the integrity of the survey data collection [79,80]. A log record will be initiated and maintained to track the study status of participants throughout the mailings of the surveys. To ensure confidentiality, participants will be assigned a code number for use on all subsequent study documentation.

The survey data will be entered into SPSS. Upper and lower limits will be set for each variable, allowing the database program to detect and highlight logical and range errors requiring correction. In order to assess data entry accuracy, 10% of case records will be randomly selected and re-entered. If this data check finds an error rate greater than 1%, the accuracy of the data will be considered unacceptable and all cases will be re-entered and re-assessed.

### Analysis

#### Hypothesis one: physicians' decisions to use specific treatments depend on their judgments of the likelihood of treatment outcomes

This hypothesis will be evaluated using data from the Type 2 surveys. After adjusting for covariates, data will be examined to determine the extent to which individual judged outcome likelihoods predict treatment decisions across the sixteen cases. For example, physicians completing the Type 2 antibiotics survey will be asked to judge the proportion of patients for whom sore throat pain would resolve by day three if they 1) were given no antibiotic, or 2) were given penicillin. By subtracting the second value from the first, we can determine the judged absolute increase in likelihood of symptom resolution due to use of the antibiotic. We will then determine the extent to which differences in these outcome likelihood judgments across cases predict differences in treatment decisions (after controlling for additional factors such as demographic characteristics, specialty, practice setting, etc). The analytic strategy for this hypothesis will rely on the use of hierarchical or mixed model regression, which permits the estimation of physician-specific coefficients and the inclusion of physician-level covariates [81-83]. For example, the analysis of the decision to treat with antibiotics could be performed using a hierarchical multivariate regression models for an individual physician, 'physician I'. This model will take the form:

TR_ij _= b_0i _+ b_1i _A_ij _+ b_2i _B_ij _+ b_3i _C_ij _+ error

where TR_ij _represents how strongly physician i feels about the patient's treatment in vignette j; b_0i _is a physician specific intercept; and A_ij _and B_ij _represent within- physician covariates.

The second level of the model will describe variation between physicians. This level will ordinarily assume that the coordinates (b_0_, b_1_, b_2_, etc.) vary at random across physicians. These coordinates measure the effect of the components of A, B, and C within physician i. We will also consider using models where the intercept and the coefficients of A, B, and C are functions of physician characteristics.

The hierarchical model will provide estimates of the physician-specific coefficients and components of variance. The more elaborate models will also provide estimates of coefficients describing inter-physician variability as a function of physician characteristics (components of specialty, practice setting, etc). The model-fitting process will use standard software for hierarchical and mixed models, including subroutines from SAS, MLWin [83] and BUGS [84].

#### Hypothesis two: physician judgments of the likelihood of treatment outcomes will sometimes be inaccurate

To evaluate this hypothesis, data from the Type 1 surveys will be used to test whether judged outcome likelihoods for a representative patient match best evidence from systematic reviews. For example, judged absolute increase in resolution of symptoms due to antibiotics use will be computed as described above (hypothesis one). This will allow the comparison of judged estimates with the 95% confidence intervals reported by these trials and tabulation of the percentage of physicians that are outside the 95% confidence intervals (i.e. maintaining beliefs that have been "ruled out" by the trials). We will display the distribution of the physicians' judgments compared to the trials' best estimate and surrounding 95% confidence intervals.

#### Hypothesis three: specific judgment heuristics can account for some of the inaccuracies of physician judgments of treatment outcomes

Type 1 surveys will include questions on whether rare or vivid outcomes had been seen by the physician in the previous year. The extent to which the answers to this question affect judgment accuracy will be analyzed using an approach similar to that for hypothesis one. Note, however, that there will only be one observation per physician, therefore hierarchical modeling will not be required. This analysis will test whether experience of and memory for rare, bizarre, or vivid outcomes (e.g. suppurative complication of a streptococcal infection) affect the assessment of the overall likelihood of such an outcome. The response variable in the regression models will be the assessment of the likelihood of outcome for the case presented in the Type 1 survey. Independent variables will include physician characteristics (e.g. demographics, specialty, and practice setting) and the physicians' recollections of rare outcomes.

#### Hypothesis four: predictable groups of physicians will be more apt to be inaccurate in their judgments of treatment outcomes

This hypothesis will be addressed using data from the Type 1 warfarin survey. The judged likelihood of outcomes for each physician will be calculated, then compared with the best evidence as indicated for hypothesis two. After controlling for a variety of covariates (age, gender, practice setting, etc.), the accuracy between the physicians' specialty groups will be compared (family physicians and internal medicine specialists). If groups differ in accuracy after controlling for the covariates, exploratory analysis will examine which decision cues could explain these differences, and whether differential reliance on these decision cues between groups explain the group differences in accuracy. These decision cues will then be further examined and purposely varied in the Type 2 survey. For example, logistical concerns about managing warfarin therapy may be more relevant to family physicians than internists (who often are not responsible for long-term management), and might therefore contribute to group differences. Systematic manipulation of this cue in the Type 2 survey would reveal whether this cue contributes to group differences in treatment decisions.

#### Hypothesis five: some judgment inaccuracies will stem from physicians overweighting cues that are unrelated to treatment outcomes, and/or underweighting cues that are related to outcomes

This hypothesis will be evaluated using data from the Type 2 surveys. The analytical approach is identical to that described in hypothesis one, with the response variable being "judged probability" instead of treatment decision. This approach is conceptually inspired by lens modeling, otherwise known as social judgment analysis [85-87]. The approach involves systematically varying the levels of several sources of information (cues) between a series of vignettes. From these vignettes, the judgment strategies employed by physicians when making their diagnoses can be inferred. This judgment strategy can be represented as a linear regression model, with standardized regression weights describing the relative importance of each cue in determining a physician's diagnosis. While the linear model does not necessarily indicate what the physician was thinking at the time of judgment, it will predict those judgments accurately [88], and indicate which cues affected judgment [89].

We will also tabulate the proportion of physicians for whom one or more of the non-predictive variables have coefficients different from zero, as assessed by the 95% posterior probability region; this implies these variables are used as predictors of either benefits or harms. We will then tabulate the proportion of physicians using each specific type of variable to make their judgments.

For all regression models, we will employ graphical approaches to look for outliers and influential observations, while statistics measuring model fit will also be calculated. Steps to control the extent of missing data items will be built into each aspect of the data collection and data management process. During the final analysis of the data we will rely on multiple imputation techniques to handle the presence of missing data elements. We will also compare the results to those obtained from the analysis based on complete cases only.

### Sample size and power

Our survey response rate estimates are based on previous similar work examining physicians' treatment decisions for patients with HIV [74]. That study involved mailing a Type 1 survey to a random sample of 2,495 physicians from the American Medical Association master file. Similar methods to those planned for the current proposal were used to enhance participation, including an honorarium of $10 per physician. Of all surveys distributed, 3.8% (96/2,495) were returned due to an incorrect address, and 2.6% (65/2,495) were returned because the physician had retired. The final response rate for the eligible physicians in this study was 51.4%. Given our plan to mail each survey to a minimum of 300 physicians, we expect 6% will be ineligible, leaving 282 eligible. Of these, we expect at least 50% will return completed surveys. Thus our expected minimum total sample size will be 141 for each survey. In the case of the warfarin surveys, we expect 141 family practitioners and 141 internal medicine specialists to respond.

Hypothesis four will involve measuring the difference in accuracy between two groups. Assuming a minimum critically important difference in accuracy of 0.5 standard deviations, the power with a type one error rate of 5% and 141 physicians per group will be 0.98. As we will likely need to adjust for some covariates in this comparison of accuracy, some allowance needs to be anticipated. Previous simulation studies have suggested that adjusted analyses should have at least 90% as much power as the unadjusted models. Thus, we can expect to have at least 88.2% power (0.9 × 0.98 = 88.2%) [90].

Hypotheses one, three, and five involve prediction both within and across physicians, but it is only in the latter case where power becomes an issue, as statistical significance of factors within a particular physician is not an important issue in this study. Drawing on sample size conventions for prediction [91] and taking physician as the observation, we have chosen to estimate the number of physicians needed on the basis of the number of degrees of freedom (df) in the covariates that need to be modelled. We propose to include gender (1 df), years of experience (2 df), practice setting (2 df), volume of relevant cases (1 df), current test ordering practice (1 df), and previous experience with rare side effects (1 df). A total of 8 df multiplied by a rule of thumb fifteen observations per degree of freedom [91] suggests we need at approximately 120 respondents; we expect 141. Hypothesis two involves determining the percent of physicians that maintain judged outcome likelihoods that have been ruled out by 95% confidence intervals from trials. The 95% percent confidence interval for the percent of physicians based on assuming maximum variance (p = .5) will be less than ± 1.96 × sqrt (0.25/141) = 0.082.

## Discussion

We see this work as a necessary prerequisite for the development and implementation of an intervention that will increase the accuracy of judged outcome probabilities and improve treatment utilization. In the next phase of this work, we will use findings from this study to develop a computerized feedback task designed to improve the accuracy of these judgments. This study will tell us the scope of the inaccuracies for our two clinical decisions, determine a number of sources of these inaccuracies, establish which physicians make which sorts of error, and allow us to determine what kinds of feedback will be most effective in improving judgment accuracy.

This work will be the first to assess in detail potential reasons for physicians' suboptimal management of two very important medical problems. It will be the first large-scale study to examine the relationship between physician-specific judgment characteristics and medical decisions for important, inappropriately treated clinical conditions. It will also be the first to examine the accuracy of outcome judgments for these clinical conditions, and to examine whether they are affected by judgment heuristics and biases.

We believe that the current proposal will have far-reaching implications. It will provide insight as to why physicians persistently use treatments inappropriately, despite clear evidence about how they should be used. More importantly, this work will lead directly to the development of focused interventions that could greatly improve treatment utilization. For instance, the development of online computer software that provides physicians with direct, immediate feedback comparing their outcome probability estimates to the best available evidence may lead to substantial improvements in judged outcome probabilities. While the question of whether such improvements lead to improved treatment behaviour must be left to a future full-scale RCT, the ground work proposed here will allow us to determine whether developing such a tool to be the focus of an RCT would be warranted.

It is likely that a wide variety of other treatment situations are also affected by inappropriate outcome estimates. For example, it is quite common to see over-utilization of expensive, invasive, and/or high technology interventions, such as percutaneous transluminal coronary angioplasty (PTCA) [92], and screening for prostate cancer with prostate specific antigen (PSA) assays [93,94], without convincing evidence of the effectiveness of these interventions. The techniques proposed here will provide a mechanism to understand the judgment processes that go into the use of these interventions, and potentially to increase appropriate use.

### Limitations

Several study limitations warrant consideration. First, the extent to which responses provided to these survey-based vignettes reflect real-world management of patients in actual practice is unclear. However, evidence is accumulating to support the validity of clinical case vignette-based research. Physician decisions in response to case vignettes generally mirror their decision making for simulated patients with the same clinical problem. Furthermore, the vignette approach approximates real-world decision making much better than does data from standard chart abstraction techniques [95-97]. We have carefully tried to maximize the validity of our vignettes by 1) using vignettes with high face validity; 2) allowing for responses similar to those one might make in practice; 3) avoiding "cueing" subjects by listing responses they are unlikely to consider in real life; and 4) avoiding suggesting which responses are expected.^97 ^We will extensively pilot test draft surveys to ensure that the vignettes are representative of real-world decisions.

There is some possibility of significant response bias, given that we have conservatively projected our response rate to be 50%. This level of responding is consistent with our experience with this type of survey [74], as well as other similar surveys [98-100], while recent systematic reviews have estimated similar overall mean response rates to physician surveys [101,102]. There is evidence that physicians who do not respond to mailed surveys are less active in and knowledgeable about the relevant clinical areas than those who do respond [103]. This might mean that our results will understate the difficulties physicians have judging outcomes of the treatment of interest, and the degree they use non-predictive variables to make these judgments. However, any such response bias would result in greater (not reduced) accuracy in judgments, and therefore reduce the likelihood of supporting hypothesis two, by yielding a conservative estimate of the extent to which these physicians make inaccurate outcome judgments.

Finally, it may be that some treatment decisions depend as much on the value or importance placed on the outcomes as they do on their likelihood. Evidence suggests this may be true of patient decision making, where the presence of vivid but rare potential side effects can have disproportionate effects on decision making [104], and may well be true of physician decision making as well. For example, we have observed that treatment differences between UK and US physicians deciding about drug therapy for seizure patients may stem from differences in the judged importance of particular side-effects. Indeed, some have argued that for physicians "value is a consideration in every decision representation" [13]. While methods of measuring the values or importance of health outcomescalled "utilities" in decision analysisexist, they are complex and time-consuming; we therefore decided to limit the scope of the current project to a consideration of judged outcome likelihood.

### Changes to the protocol after funding

This protocol has been peer-reviewed and approved for funding by the Canadian Institutes of Health Research, and has ethics approval from the Ottawa Hospital Research Ethics Board. Our original proposal targeted use of antibiotics for sore throat, and the use of HMG Co-A reductase inhibitors (statins) for coronary artery disease (CAD) and hypercholesterolemia. When detailed planning began after funding was received, the literature on use of statins for CAD had grown more complex; it was less clear whether statins are universally under-used, or rather under-used in some populations and over-used in others. This increasing complexity would have required us to focus on a specific patient subgroup, making it more difficult to find physician respondents that deal with the specific group. We therefore decided to focus on anti-coagulants for AF instead; methodology and analysis has not changed.

## Abbreviations

ACE Angiotensin-converting enzyme

ADR Adverse drug reactions

AF Atrial fibrillation

CAD Coronary artery disease

CHF Congestive heart failure

CIHR Canadian Institute of Health Research

CRTN Canadian Research Transfer Network

Df Degrees of freedom

HIV Human immunodeficiency virus

KT Knowledge translation

MI Myocardial infarction

PSA Prostate specific antigen

PTCA Percutaneous transluminal coronary angioplasty

RCT Randomized control trial

## Competing interests

The author(s) declare that they have no competing interests.

## Authors' contributions

RP conceived the general research questions. JB and RP wrote the proposal. RP, MH, KS, EB, and JG provided specific clinical and/or methodological expertise. AL and JB wrote the protocol and methodology. All authors contributed to the development of the specific research questions, reviewed the proposal and protocol, and read and approved the final manuscript.
